# First *WNK4*-Hypokalemia Animal Model Identified by Genome-Wide Association in Burmese Cats

**DOI:** 10.1371/journal.pone.0053173

**Published:** 2012-12-28

**Authors:** Barbara Gandolfi, Timothy J. Gruffydd-Jones, Richard Malik, Alejandro Cortes, Boyd R. Jones, Chris R. Helps, Eva M. Prinzenberg, George Erhardt, Leslie A. Lyons

**Affiliations:** 1 Department of Population Health and Reproduction, University of California Davis, Davis, California, United States of America; 2 The Feline Centre, University of Bristol, Langford, Bristol, United Kingdom; 3 Centre for Veterinary Education, University of Sydney, Sydney, Australia; 4 Institute of Veterinary, Animal & Biomedical Sciences, Massey University, Palmerston North, New Zealand; 5 Molecular Diagnostic Unit, University of Bristol, Langford, Bristol, United Kingdom; 6 Institute of Animal Breeding & Genetics, Justus Liebig University, Giessen, Germany; IGBMC/ICS, France

## Abstract

Burmese is an old and popular cat breed, however, several health concerns, such as hypokalemia and a craniofacial defect, are prevalent, endangering the general health of the breed. Hypokalemia, a subnormal serum potassium ion concentration ([K^+^]), most often occurs as a secondary problem but can occur as a primary problem, such as *hypokalaemic periodic paralysis* in humans, and as *feline hypokalaemic periodic polymyopathy* primarily in Burmese. The most characteristic clinical sign of hypokalemia in Burmese is a skeletal muscle weakness that is frequently episodic in nature, either generalized, or sometimes localized to the cervical and thoracic limb girdle muscles. Burmese hypokalemia is suspected to be a single locus autosomal recessive trait. A genome wide case-control study using the illumina Infinium Feline 63K iSelect DNA array was performed using 35 cases and 25 controls from the Burmese breed that identified a locus on chromosome E1 associated with hypokalemia. Within approximately 1.2 Mb of the highest associated SNP, two candidate genes were identified, *KCNH4* and *WNK4*. Direct sequencing of the genes revealed a nonsense mutation, producing a premature stop codon within *WNK4* (c.2899C>T), leading to a truncated protein that lacks the C-terminal coiled-coil domain and the highly conserved Akt1/SGK phosphorylation site. All cases were homozygous for the mutation. Although the exact mechanism causing hypokalemia has not been determined, extrapolation from the homologous human and mouse genes suggests the mechanism may involve a potassium-losing nephropathy. A genetic test to screen for the genetic defect within the active breeding population has been developed, which should lead to eradication of the mutation and improved general health within the breed. Moreover, the identified mutation may help clarify the role of the protein in K^+^ regulation and the cat represents the first animal model for *WNK4-associated* hypokalemia.

## Introduction

Potassium is the most abundant cation in mammals [1], [2]. The resting membrane potential of cells is affected by the relationship between intracellular and extracellular potassium concentrations and the resting potassium conductance [3]. Since the extracellular potassium greatly affects the tendency of cells to fire action potentials, potassium plays a crucial role in the function of nervous tissue and muscle (skeletal, cardiac and smooth) throughout the body [1], [2], implying perturbations can be debilitating or even life-threatening. To maintain ideal body homeostasis, potassium excretion and dietary intake must be balanced [4]–[6]. Abnormalities of potassium homeostasis can occur as a primary condition, or as a secondary disorder [7]–[10]. Inherited hypokalemia has been discovered in a variety of mammals by genetic studies of individuals and families affected by clinical disease. A classic syndrome of myopathic weakness, *hyperkalemic periodic paralysis* (*HYPP*), has been defined genetically in humans [11]–[14] and horses [15]–[18]. Genetic studies have shown that in humans, *HYPP* is attributable to a channelopathy associated with abnormal sodium conductance, usually inherited as an autosomal dominant trait. Primary hypokalemic disorders have been documented in humans, manifesting as episodic weakness associated with low serum potassium [19].

*Feline hypokalaemic periodic paralysis* or *Burmese hypokalaemic periodic polymyopathy (BHP)* has been recognized since the seminal cases described by Blaxter and colleagues [20]. The disease is characterized by muscle weakness associated with intermittent hypokalemia [21]. Genetic studies suggest an autosomal recessive condition in Burmese cats [20]. Blaxter [20] provided a full description of the condition in the Burmese cat breed of the United Kingdom, Jones and collaborators recorded similar findings within Burmese cats from New Zealand [22], while Mason and Lantinga documented the condition in cats in Australia and the Netherlands, respectively [23], [24]. BHP has also been diagnosed elsewhere in Europe [15], however the disease has not been diagnosed within the Burmese cat population within the United States.

Classically, signs of BHP are episodic but in some cats, the weakness is incessant. During an episode, muscle pain (myalgia) from palpation can be a prominent sign. Cats can present with severe generalized muscle weakness, although more commonly weakness of the cervical muscles as evidenced by ventroflexion of the head and neck, head bobbing and dorsal protrusion of the scapulae [20], [23], [25]–[27]. The gait becomes short and maximal recruitment of motor units gives rise to muscle tremor. Cats with more generalized weakness have a crouching gait, especially evident in the hind limbs. Clinical signs of hypokalemia are thought to develop once serum potassium concentrations decreases below 3.0 mmol/l, although the rate with which the potassium concentration decreases and the lowest concentration achieved during an episode probably also influence the onset and the severity of signs. Although the course of the disease is usually affected by veterinary intervention, including potassium supplementation, serum potassium usually spontaneously normalizes after an episode. The disease usually becomes evident when kittens are two to six months of age, although some cases have not been detected until 2 years of age. Clinical signs may be triggered by stress or exercise. In some patients, the condition improves spontaneously or with therapy over a period of time, with some cats eventually not requiring on-going medication. However, some cats with BHP have life-time requirements for a potassium supplementation.

A genome-wide association study (GWAS) using the illumina Infinium feline 63K iSelect DNA array was performed on hypokalemic and healthy Burmese to localize the gene responsible for the hypokalemic periodic polymyopathy. A significant association was identified on cat chromosome E1, which led to a mutation discovery in *WNK4*. A genetic test has been developed and carrier cats can be easily identified prior to mating, avoiding the generation of affected cats. The *WNK4* mutation in the Burmese cat is linked for the first time with hypokalemia in a mammalian species; a novel model for human disease.

## Results

### Clinical Presentation

**Figure 1:**
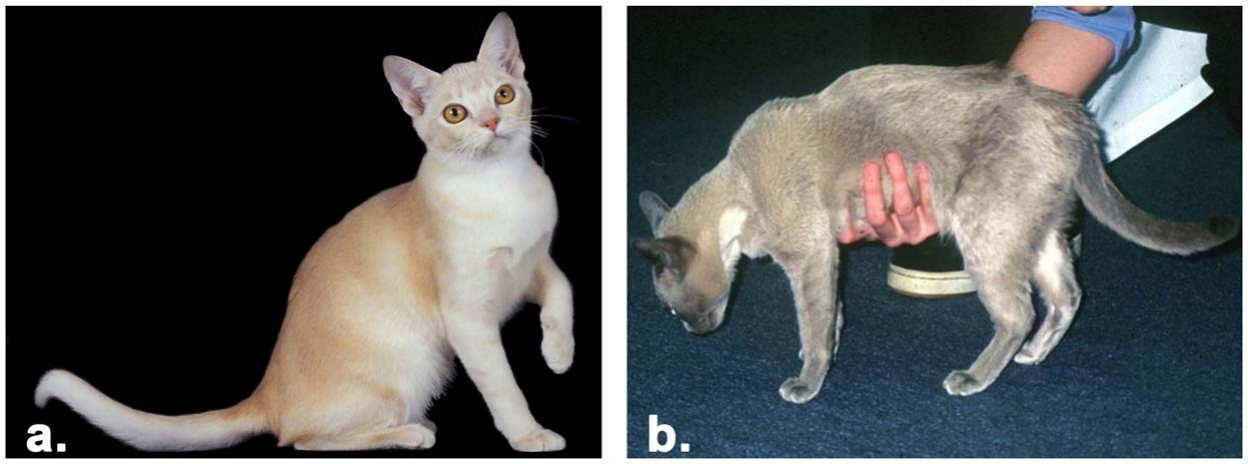
Phenotypes of Burmese cats and Burmese affected with hypokalemia. **a.** Unaffected Burmese of champagne coloration, **b.** hypokalemia affected Burmese of red coloration, displaying ventroflexion of the head and neck.

Clinical presentation of cats diagnosed with hypokalemia included: episodic or incessant musculoskeletal weakness frequently characteristically by ventroflexion of the head and neck (**Figure 1**). Myopathic weakness was additionally associated with a stiff/stilted gait, muscle tremor, head bobbing and dorsal protrusion of one or both scapulae. Some cats were thought to have myalgia on the basis of pain on muscle palpation or a shifting lameness, which resolved with oral potassium supplementation. In some cats, a weaving or a crouching gait, hypermetric forelimbs, a wide-based hind-limb stance were also observed.

Most cats were diagnosed when young adults, between two and six months of age, although occasional cats were diagnosed at a later age. Although all cats considered as having hypokalemia had reduced [K^+^] (by definition) at some point in time, this was not always a consistent finding in affected cats, and sometimes it was necessary to collect sequential blood samples to identify the time when cats were unequivocally hypokalemic. Suggestive signs in Burmese cats and sometimes increased serum CK or aspartate aminotransferase (AST) activities were helpful in focusing the clinicians’ attention on the possibility of a myopathy. The demonstration of overt hypokalemia was further substantiated by demonstrating an improvement in muscle strength after oral potassium supplementation with potassium gluconate or chloride, with a concurrent return of serum muscle enzyme activities to the reference range.

### Genome-wide Association Study

**Figure 2:**
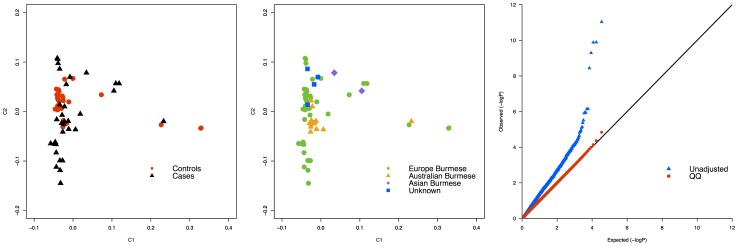
Multi-dimensional scaling and QQ-plot of Burmese cats analyzed for hypokalemia. **a.** Multi-dimensional scaling showing the distribution in 2 dimensions of the cases and controls of Burmese cats. The black asterisk indicates the location of the two random bred cat outliers excluded from the association analyses. **b.** Multi-dimensional scaling showing the differences between the geographical origins of the cats included in the analyses. The black asterisk indicates the 10 European Burmese cases that did not cluster with the majority of the Burmese samples included in the study. **c.** Q–Q plot showing an inflation in the test statistics with a λ = 1.8.

The affected Burmese cats originated from Australia, UK and Germany (samples from both countries were grouped in the Europe category), therefore unaffected, control cats were selected from the same countries with an established minimal relationship to the cases (**Table 1**). Seventy-two cats, including 38 cases (36 Burmese and 2 random bred cats) and 34 controls, were submitted for SNP array genotyping. Ten cats, including 1 case and 9 controls, had genotyping rates <90% and were excluded from analysis, but included in the cohort of samples that were genotyped for the identified mutation (**Table 1**). Testing for stratification by MDS revealed that the cats were not in one genetic cluster **(** **Figure 2a**). Even though the distribution of the cases and controls was not equally dense across the cluster, only two obvious affected outliers, the random bred cats, were identified and excluded from the analysis, thus the final number of cases included in the analyses was 35 Burmese. Additionally, 10 cases from the same geographical region, European Burmese, did not group together within the cluster (**Figure 2b**). Twelve samples had a 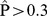 and the two random bred samples had a 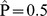 suggesting that the two samples are sibs. The detected genomic inflation factor (λ ) was 1.8 (**Figure 2c**). To reduce λ, several case-control studies were conducted after several samples pruning (**Figure S1**). The sample size range from 26–50 while the p-value varies from 7.22×10^−5^–1.66×10^−9^. The highest λ is detected when none of the Burmese samples is excluded from the analyses. The lowest λ = 1.09 is detected when the most genetically similar control is selected for each case (**Table S2**).

**Table 1:** Domestic cats genotyped for the mutation associated with hypokalemia.

Breed	Origin	Phenotype	No.		Genotype	
				C/C	C/T	T/T
	USA	Normal	16	16	0	0
	Asia	Affected*	2	0	0	2
Burmese	Europe (UK	Affected* +	22	0	0	22
	+ Germany)	Normal+	16	10	6	0
	Australia	Affected*	8	0	0	8
		Normal	0	0	0	0
Random Bred	UK	Affected*	2	0	0	2
Pedigree	Europe	Affected*	9	0	0	9
		Normal**	26	10	16	0
Bombay	USA	Normal	13	13	0	0
Random Bred	USA	Normal+	10	10	0	0
Tonkinese	USA	Normal	10	10	0	0
Total			134			

* All available affected cats were used in GWAS except only four of nine cats in the pedigree (Figure 2).

** From the pedigree, only two of 26 cats were selected for the study (Figure 2).

+ Cats used for sequencing: two affected, two controls, one random bred.

**Figure 3:**
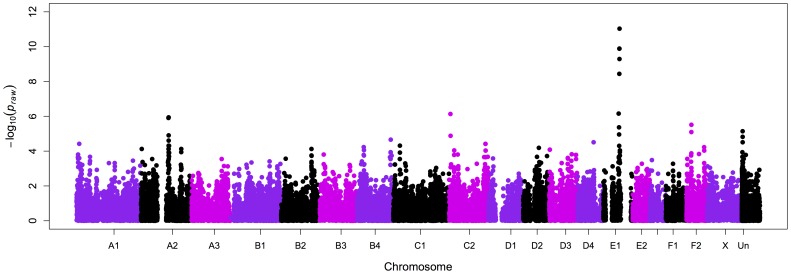
Manhattan plot summarizing the case – control GWAS for Burmese cats with hypokalemia. The associated SNPs from 72 to 73 Mb on cat chromosome E1 (chromosome 14) obtained by analyzing 35 cases and 25 controls, correspond to a region containing the two candidate genes, *KCNH4* at ∼570 Kb and *WNK4* at ∼1.2 Mb from the SNP with the highest association (E1.73054644, *P* raw = 9.274×10^−12^ uncorrected).

After evaluating the genotype qualities of the 62,897 SNPs on the array, 34,800 markers passed quality control, 27,152 SNPs had a MAF <5% and 1206 SNPs a GENO value >0.1. By analyzing 35 cases and 25 controls, a significant association was identified with several SNPs on chromosome E1 (**Figure 3**), with the most significant association for a SNP at position 73,054,644 (the Whole Genome Shotgun project has been deposited at DDBJ/EML/GenBank under the accession AANG00000000. The sequence assembly used in this study is the second version, AANG02000000). A second association was detected with chromosome C2 with a SNP at position 5,583,090 (**Table S2**). After correcting *P*_raw_ values for multiple hypotheses testing by permutation, six SNPs on chromosome E1 reached genome wide significance (*P*_genome_ = 0.00002 for SNP E1 at position 73,054,644) and one less significant SNP on chromosome C2 position 5,583,090 (*P*_genome_ = 0.046) (**Figure S1**). The strongest association on chromosome E1 was detected within a region between chromosome positions ∼72 to 73 Mb. A haplotype block was identified from position 73,054,644 to position 74,507,499 with a frequency of 75.7% across all cases, suggesting the most likely region associated with the disease (**Figure S3**). Within the same block, controls showed a 16.6% haplotype frequency. The two affected random bred samples excluded from the case-control study, showed a different haplotype compared to the affected Burmese cats. Inspection of genes within this haplotype block revealed two strong candidates involved in potassium level regulation, *KCNH4* at ∼570 Kb and *WNK4* at ∼1.2 Mb from the SNP with the highest association (**Figure S4**). The location of the two genes compared to the SNP position is presented in Figure S4. Since *WNK4* is not annotated in the *Felis catus* genome, the distance between the two genes (*KCNH4* and *WNK4*) in the human genome was used to infer the distance between the same genes in the cat assembly.

### KCNH4 and WNK4 Analyses

A partial *KCNH4* (GenBank accession no. JQ522970) coding sequence (CDS), which was missing exons 1 and 13, was analyzed in four unrelated Burmese cats (two cases and two controls) and one random bred cat. Four variants were identified across the CDS and none were concordant with the phenotype (**Table S3**). Polymorphisms identified in the intronic sequence are reported in **Table S3**.

The entire CDS of *WNK4* (GenBank accession no. JQ522971), including partial 5′ UTR and the 3′ UTR, was analyzed in the same four Burmese (2 cases and 2 controls) and a control random bred cat. In humans, *WNK4* has one isoform and the length of the coding region within the transcript is 3,732 bp. In the cat, *WNK4* has 19 exons, the boundaries were confirmed by genomic sequencing of the five cats used for the analyses of *KCNH4*, producing a 3,711 bp CDS that translates into 1236 amino acids. The differences between the two transcripts are located in exon 1 (3 bp), exon 13 (3 bp) and exon 14 (12 bp+3 bp). The average CDS identity between human and cat is 89.3% and at the protein level *WNK4* is 90% identical to human. Three of the four identified exonic polymorphisms are missense mutations (**Table 2**), the affected cats showed an AGTC haplotype and the controls an AGCT haplotype at the mutated positions. The first (c.340G>A) and second (c.2851G>A) result in alanine to threonine substitutions, the third mutation (c.2899C>T) causes a premature stop codon (CAG>TAG). Moreover, the mutation is validated by the RNA transcript sequence obtained from the affected Burmese cat blood. This mutation responsible for a premature *WNK4* protein truncation lacking the C-terminal coiled coil domain and the Akt1/SGK phosphorylation site was further investigated. Several variants identified in the intronic regions (**Table 2**) were highly variable and were not concordant with the disease phenotype in the cats analyzed.

**Table 2:** SNP analyses of *WNK4* in cats with and without hypokalemia.

Breed	Phenotype	c.340 G>A	c.285 G>A	c.2899 T>C	c.3516 T>C	−20E6 C>G	+37E6 T>C	+32E9 A>G	−80E11 G>−	−64E11 G>−	+92E19 G>A	+154E19 A>G
Burmese	Case	A	G	T	C	C	T	A	C	G	A	G
Burmese	Case	A	G	T	C	C	T	A	C	G	A	G
Burmese	Control	A	G	C	T	T	T	R	Y	G	R	R
Burmese	Control	A	G	C	T	T	T	A	C	G	G	A
Random Bred	Control	G	R	C	T	T	T	A	C	G	G	A
Trace	**−**	G	G	C	T	T	C	G	T	**−**	G	A
**AA**	**change**	A -> T	A -> T	Q -> X	**−**	**−**	**−**	**−**	**−**	**−**	**−**	**−**

### WNK4 Genotyping Analyses

**Figure 4:**
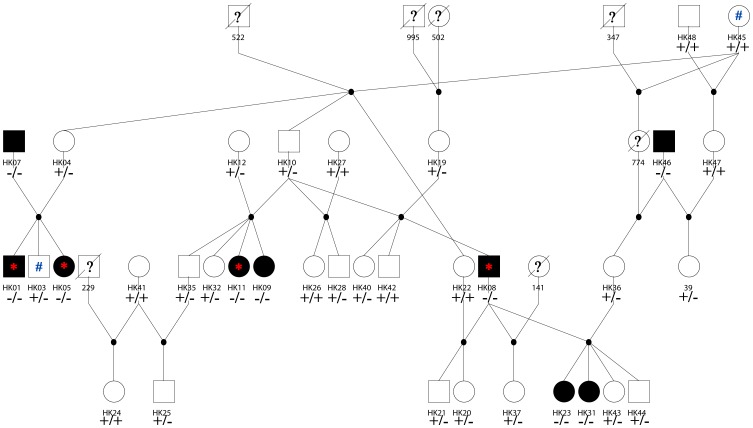
Burmese cat pedigree segregating for hypokalemia. Circles represent females and squares represent males. Black symbols indicate cats with classical clinical signs for hypokalemia, white symbols indicate healthy cats. The +/+, +/−, and −/− signs indicate cats that tested homozygous wildtype, heterozygous, and homozygous affected for hypokalemia, respectively. Cats with a question mark in the symbol indicate that the phenotypic data was not available; symbols with a diagonal line indicate that no DNA sample was available. The asterisk indicates the four affected cats included in the GWA study, the number sign (#) indicate two of the controls used for the GWA study.

A multi-generational pedigree consisting of 35 cats, segregating for hypokalemia (**Figure 4**), was genotyped for eight STRs [28]. All the relationships between cats were confirmed as reported (data not show). Nine affected cats, with either carrier or affected parents, were confirmed by genotyping as homozygous for the c.2899C>T mutation. Of the 26 unaffected cats, ten were homozygous wildtype and 16 were carriers.

All the unaffected cats from other USA breeds (Bombay and Tonkinese), as well as random bred cats, were homozygous wildtype (n = 33). Fifty Burmese cats from Asia, Australia and Europe (Germany and UK), including 34 GWAS affected and 16 unaffected cats, with available potassium concentrations were genotyped and all the affected cats were homozygous for the c.2899C>T mutation; the unaffected individuals were either carriers or homozygous wildtype (**Table 1**).

A cohort of 268 cats from different breeds including Asian, Australian Mist, Burmese, Burmilla, Tiffanie and 2 unknown cats was used to estimate the disease allele frequency. The frequency of the affected allele within the population is estimated as 14.9% (**Table S4**).

## Discussion

Variations in the flux and balance of several ions, such as K^+^, Ca^2+^, Na^+^, and Cl^−^ play key roles in specific tissue functions. In skeletal muscle, perturbations of these electrolytes are responsible for periodic paralyses, paramyotonia, and myotonia [1], [29]. Periodic paralyses constitute a group of hereditary muscle disorders characterized by acute and reversible attacks of muscle weakness associated with decrease blood pressure [30] in humans and is reported in other species such as, cow [31], horse [32], [33], rat and dog [34], [35]. Hypokalemic periodic paralysis is the most frequent form of periodic paralysis in humans, with an estimated prevalence of 1/100,000 [36]. Death due to paralysis of respiratory muscles [37] or from cardiac arrhythmia, secondary to a severe decrease in blood potassium concentration has been reported [38].

This case-control genome-wide association study was performed on a cohort comprising of 35 cases and 25 controls from the Burmese breed. Although the disease is usually episodic, definitive diagnosis can be made by the clinical presentations. Polymyopathy was reported in the domestic cat in 1983 [25] and hypokalemic periodic paralysis in the Burmese breed was first reported the UK and later identified in Australia and New Zealand [20], [22], [25], [26], [39]. However, Burmese cats in the USA have not been reported with hypokalemia. Due to differences in breed standards and health concerns, the Burmese in the USA are generally isolated from the Burmese in other countries. For the non-American-derived Burmese, diseases endangering the general health of the breed are hypokalemia [20], [25], diabetes mellitus [40] and an orofacial pain syndrome [41], [42]. The American-derived Burmese are highly associated with a craniofacial defect [43] and flat-chested kitten syndrome [44] is found in both breed populations. Amongst cat breeds, both the USA and non-USA Burmese populations present the highest linkage disequilibrium within cats (data not shown), likely due to geographical isolations and population bottlenecks. Due to these suspected population differences, USA-derived Burmese were not used as controls. In addition, 27,152 SNPs (43.1%) were excluded from the analysis due to MAFs <0.05, suggesting low genetic diversity in the breed, which was previously suggested by microsatellite analyses [45]. All the non-American Burmese clustered to some extent, indicating that some population sub-structure is present, which was supported by a genomic inflation factor of 1.8. The genomic inflation was reduced after each sample pruning, and got as low as 1.09 after excluding 34 samples. However, the lower samples size reduces power in detecting the association. When the samples size drops as low as 34, the SNP previously detecting the association is no longer the most significant association and the regional candidate would not be identified. To reduce genomic inflation, sib-pairs from a litter would be more ideal, however, as with most disease ascertainment, cases were collected over a long period and access to normal sibs or parents was not usually feasible.

The haplotype analysis of the 2.6 Mb region surrounding the most significant SNP association identified a 1.45 Mb block, with the most common haplotype having a frequency of >75% within the cases and ∼16% in the controls, supporting the region containing *KCNH4* and *WNK4* as strongly associated with the disease. The two related random bred cats were excluded from the association analyses, both were outliers in the MDS analyses and both had a completely different genetic background surrounding the associated SNPs as determined by the haplotype analysis. However, the random bred cats were homozygous for the identified hypokalemia mutation in the Burmese breed suggesting a mutation that precedes breed information. By fine mapping, a common short haplotype block with the controls would probably be identified.

Within ∼1.45 Mb of the highest associated SNP, two viable candidate genes, *KCNH4* and *WNK4*, were identified. In *KCNH4*, no polymorphisms were identified between cases and controls, suggesting no differences between the affected and wild type alleles. However, sequencing of *KCNH4* was not completed, hence the gene cannot be excluded as responsible for the disease. Sequencing of the second candidate gene, *WNK4* identified a c.2899C>T nonsense mutation in the coding sequence. To confirm the association of the c.2899C>T mutation with phenotype, a multi-generational pedigree segregating for the disease was genotyped. All the affected cats tested homozygous for the premature stop codon, while the unaffected Burmese were either wildtype or heterozygous, supporting the c.2899C>T mutation as likely responsible for the disease. All identified cases in the study were homozygous for the mutation, while control Burmese were either heterozygous or wildtype. All available parents of the affected cats were heterozygous for the identified mutation. The genotyping was extended to a limited random bred population from South Asian and other South Asian - derived breeds, such as Bombay and Tonkinese, which use or have used Burmese in their breeding programs. The mutation was not identified within these cats. The initial estimate of the mutation frequency within the non-USA derived Burmese population, as well as in the breeds that use Burmese in outcross programs, is 14.9%. The frequency was obtained using data from a service laboratory from cats submitted for hypokalemia genetic testing, hence likely suspected to be at risk for disease, thus a positive bias in the data is expected. The genetic test will greatly assist breeders to avoid mating that could generate affected cats and identify carrier cats that could be excluded from the breeding program.

Point mutations in *WNK4* have been associated in humans with the dominant disorder familial hyperkalemic hypertension and characterized by dysfunction in renal Na^+^, K^+^, and Cl^−^ homeostasis [46]–[52]. Wilson et al. [50] originally identified *WNK4* as a serine-threonine protein kinase that is expressed virtually exclusively in the kidney [53], localizing to the distal convoluted tubule and the cortical collecting duct [54], adjacent segments of the distal nephron that play a key role in salt, water, potassium, and pH homeostasis [50]. *WNK4* is a member of the WNK kinases that have recently become a major focus of investigation because they comprise a previously unrecognized signaling pathway. The pathway appears to be essential for normal development, regulation of arterial pressure, normal electrolyte balance and for sensory nerve function, however the mechanisms by which WNK kinases regulate these processes are poorly understood [48], [49].

The *WNK4* gene in humans is 16 Kb, contains 19 exons [50] and the protein is characterized by a kinase domain, an auto inhibitory autophosphorylation domain that lies in close proximity to the C-terminal end, two coiled-coils domains and proline-rich sequences [46]. The truncated protein in affected Burmese lacks the C-terminal coiled-coil domain, and is missing the highly conserved Akt1/SGK phosphorylation site at position S1169 [54]. Wang et al [55] demonstrated that truncated WNK4 constructs lacking the C terminus had no kinase activity toward themselves (autophosphorylation), hence *WNK4* is constantly active. Studies suggest that when WNK4 is over-expressed, blood pressure is lower and when challenged with a low potassium diet, serum K^+^ is also reduced [56]. To date, studies of blood pressure in affected Burmese cats with hypokalemia have not been undertaken; it would therefore be informative to determine if cats are hypotensive during episodes of hypokalemia. Morphological studies in mice show that extra *WNK4* copies reduced the abundance of the thiazide-sensitive Na-Cl cotransporter (NCC) in the kidney cortex [47], [57], [58]. The interaction between WNK4 and NCC, through its kinase domain, is suggested to result in high lysosomal degradation of NCC. When WNK4 is inhibited by autophosphorylation, NCC transfers to the membrane and Na^+^ and Cl^−^ re-absorption activity is initiated. The deranged physiology seen in cats with BHP may be similar to the loss of function of NCC in humans [59] and mice [60], [61]. In hypokalemic cats, the *WNK4* protein should be constantly active since the autophosphorylation site is missing and the protein constantly acts as an inhibitor for the NCC channel. When the NCC channel is not present on the cell surface, sodium gets reabsorbed by the epithelial channel (ENaC) [62], leaving a lumen Cl^−^ negative potential that causes a secretion of K^+^ by the ATP-sensitive epithelial K^+^ channel (ROMK) channel [63]. The presumptive role of the C-terminal region of WNK in the cat can be proven and elucidated by functional studies using truncated transcripts in the future.

## Materials and Methods

### Animal Screening and Clinical Description

Thirty-eight cats with a diagnosis of hypokalemia were ascertained (**Table 1**, **Figure 1**). Most cases were diagnosed by the authors or by close colleagues; however, some were obtained by solicitation and submitted by owners who had received a BHP diagnosis for their cat from their primary care veterinarian. Burmese cats were verified as having BHP based on consistent clinical signs, seen in temporal association with serum potassium less than 3.0 mmol/l, and usually in concert with elevated creatine kinase (CK) activity in serum or plasma. Furthermore, all cats suspected of having hypokalemic polymyopathy had an obvious improvement in muscle strength with oral potassium supplementation (using potassium gluconate [4 mmol every 12 hours] or potassium chloride [3.5 mmol every 12 hours]). Unaffected cats (n = 34) had no clinical signs or owner/breeder reported evidence for BHP. No IACUC approvals were obtained for this study since samples were retained from patients as part of their primary care for disease diagnosis. Standards of veterinary care appropriate for an individual cat’s health and conditions were determined by the primary care veterinarian.

### Genotyping

Thirty-eight cases (36 Burmese and 2 random bred cats) and 34 control Burmese were submitted to genotyping analysis. Whole blood samples were collected from affected cats as part of establishing their clinical diagnoses. Buccal swabs were donated for some affected and unaffected cats by Burmese breeders and owners. Moreover, a pedigree of cats segregating for hypokalemia was developed from a sub-set of submitted samples (four were included in the GWAS) (**Figure 4**). Additional unaffected cats were selected from an available database of non-USA origin/domiciled Burmese cats. Genomic DNA was isolated from blood and buccal swab samples using the DNAeasy Kit (Qiagen, Valencia, CA) and concentrated using the Genomic DNA clean & Concentrator Kit (Zymo Research, Irvine, CA) when necessary. Quality and quantity of DNA was confirmed by visualization with agarose gel electrophoresis and by optical density using an UV exposure. Approximately 600 ng of genomic DNA was submitted to Neogene, Inc. (Lincoln, NE, USA) for genotyping on the illumina Infinium Feline 63K iSelect DNA array (illumina, Inc., San Diego, CA).

### Genome Wide Association Study

SNP genotyping rate and minor allele frequency (MAF) was calculated using PLINK [64]. SNPs with a MAF <5%, genotyping rate <90%, and individuals genotyped for <90% of SNPs were excluded from further analyses. A classic multi-dimensional scaling (MDS) with 2 dimensions was performed on 56,811 SNPs in PLINK to evaluate population substructure within cases and controls. Inflation of p-values was evaluated by calculating the genomic inflation factor (λ) and was assessed with a quantile-quantile plot (Q-Q plot). A case-control association analysis was performed and corrected with 50,000 t-max permutations (-mperm 50,000). T-max permuted p-values were considered genome-wide significant at p<0.05. A Manhattan plot of the results was generated using HAPLOVIEW [65]. Linkage disequilibrium (LD) and haplotypes were determined considering SNPs from position 71,917,552 to position 74,507,499 of cat chromosome E1 and presented as plots produced by HAPLOVIEW [65]. The P (proportion of identical by state – IBS) for each individual was calculated using PLINK. To evaluate and reduce λ several strategies were applied, including, (i) exclusion of individuals with a proportion of IBD >0.3, (ii) selection of samples tightly clustered from the MDS plot and (iii) selection for each case to the closest control using the values from the MDS dimensions (**Figure S1**). After each correction, the number of cases and controls included in each analysis, as well as the p-value of the highest associated SNP, was evaluated and reported in **Table S1**.

### KCNH4 Genomic Analyses

The genomic analyses of *KCNH4* were conducted on genomic DNA from five cats including two cases and two controls from the Burmese breed and a random bred cat. Partial CDS of *KCNH4* is publicly available (http://ensembl.org) and can be found on GeneScaffold_2463∶456,252-473,070. Missing exonic sequence was retrieved by aligning the individual human exons for *KCNH4* (NM_012285.2) in the trace archive databases for *Felis catus* – WGS (http://blast.ncbi.nlm.nih.gov). Primers were designed in both UTRs and intronic regions, flanking the exons. Primers were tested for efficient product amplification on a DNA Engine Gradient Cycler (MJ Research, GMI, Ramsey, MN) and the final PCR magnesium concentrations, annealing temperatures, and amplicon sizes for each primer pair are shown in **Table S5**. PCR and thermocycling conditions were conducted as previously described [66]. The PCR products were purified with ExoSap (USB, Cleveland, OH) per the manufacturer’s recommendations and directly sequenced using the BigDye terminator Sequencing Kit v3.1 (Applied Biosystems, Foster City, CA). Sequences were verified and aligned using the software sequencer version 4.10 (Gene Codes Corp., Ann Arbor, MI).

### WNK4 Genomic and mRNA Analyses

The genomic analysis of *WNK4* was conducted as described for *KCNH4. WNK4* was not annotated in the available cat genome assembly (Dec. 2008 NHGRI/GTB V17e/felCat4) and no sequence was publicly available. The gene was identified by comparison to the homologous region on human chromosome 17 corresponding to the associated cat region [67], [68]. Cat exonic sequences were retrieved from the cat trace archive using the individual exons of human *WNK4* (NM_032387.4). The final PCR magnesium concentrations, annealing temperatures, and amplicon sizes for each primer pair are shown in **Table S5**. The PCR cycle was as follow: initial denaturation at 94°C for 4 min followed by 35 cycles as described: 94°C×30 sec, 65°C×30 sec, 72°C×30 sec and final extension at 72°C for 20 min.

Total RNA was extracted using the PAXgene™ (Qiagen) from whole blood from a control random bred cat and four affected Burmese cats from the UK. Complementary DNA templates were synthesized using SuperScript III (Invitrogen, Carlsband, CA) by reverse transcription of 1 µg of total RNA with gene specific primers (**Table S5**) and PolyT to obtain partial 5′ UTR, CDS and full 3′ UTR. Each cDNA sample was subjected to PCR using primers (10 µM each) combined as follow: 5utrF - 5utrR, F1 - R1, F2 - R2, F3 - R3, F4 - R4, F5 - R5, F6 - R6, F7 - R7, F8 - polyT. The PCR conditions were: 1.5 mM Mg, 2 µl of cDNA in a total volume of 20 µl. The PCR cycle was: initial denaturation at 94°C for 4 sec followed by a first set of 5 cycles at: 94°C×30 sec, 70°C×30 sec, 72°C×30 sec, 5 additional cycles at: 94°C×30 sec, 68°C×30 sec, 72°C×1 min and a set of 30 additional cycles at: 94°C×30 sec, 68°C×30 sec, 60°C×1 min and a final extension at 72°C for 20 min. The PCR products with appropriate lengths were purified using the ExoSap (USB) enzyme per manufacturer’s recommendations. Purified genomic products were directly sequenced in both directions using BigDye Terminator Sequencing Kit v3.1 (Applied Biosystems), purified with Illustra Sephadex G-50 (Ge Healthcare, Piscataway, NJ) according to manufacturer’s recommendations and electrophoretically separated on an ABI 3730 DNA analyzer (Applied Biosystems).

### WNK4 Mutation Genotyping

To implicate the identified mutation as causative for hypokalemia, a pedigree segregating for hypokalemia with 35 available cats, was genotyped by direct sequencing (**Figure 4**). Relationships within the pedigrees were confirmed with a published microsatellite-based parentage panel available for the cat (data not shown) [28]. Additional cats were also genotyped by direct sequencing to support correlation with disease, including 16 USA-derived Burmese, 16 unrelated unaffected, 34 affected GWAS Burmese (Asia, Europe, Australia), 2 affected random bred GWAS cats, 10 Tonkinese, 13 Bombay and 10 random bred cats (**Table 1**). Primers for the direct sequencing genotyping are presented in **Table S5**.

### Frequency Estimation

Owners submitted buccal swab samples of cats (n = 268) to determine status of the hypokalemia mutation to Langford Veterinary Services, UK (**Table S4**). Cats were genotyped for the *WNK4* mutation to establish the allele frequency in the breeding population and to further confirm association with disease. Briefly, DNA was isolated from buccal swabs as previously described [69]. The *WNK4* mutation was amplified by PCR; 12.5 µl 2× GoTaq PCR Master Mix (Promega, UK), 200 mM each Hypo For and Rev primers (**Table S5**) and 2****µl gDNA in a total volume of 25 µl using a Bio-Rad MJ Mini (Life Science, Hertfordshire, UK) as described: 95°C for 2 min, 38 cycles of 95°C×15 sec, 58°C×30 sec by 35 cycles. Amplicons were subjected to pyrosequencing using sequencing primer Hypo Seq on a PyroMark Q24 (Qiagen) using Pyro Gold reagents as described by the manufacturer.

## Supporting Information

Figure S1 **MDS plots illustrating the samples included in each case-control association analyses of cats with hypokalemia.** MDS plots showing **a.** the distribution of all the genotyped samples, **b.** the samples included in the case-control after exclusion of the related Burmese cats (p_hat >0.3), **c.** the distribution of tightly clustered Burmese samples, **d.** allowing more diversity than the cluster illustrated in (c), **e.** the closest control for each case, and **f.** the samples remaining after exclusion of related Burmese and outliers.
(TIF)

Figure S2 **Manhattan plot of Burmese GWAS for hypokalemia.** After correcting for multiple testing (50,000 permutations), five SNPs (four on chromosome E1 and one on chromosome C2) retained significant association (p<0.05; strongest SNP association, cat chromosome E1 position 73,054,644). The dashed line indicates genome wide significance.
(TIF)

Figure S3 **Haplotype analyses of cats in hypokalemia GWAS.** Presented is the area within ∼2.5 Mb of the highest associated SNP on chromosome E2 in the Burmese cases and controls. **a.** Cases haplotype block of the region surrounding the highest hit in the Burmese. **b**. From the highest hit, a block extending for ∼1.45 Mb (9 SNPs) was identified **c**. Cases haplotype frequencies, the red rectangle indicates the haplotype most frequently found within the affected cats. **d**. Controls haplotype block of the same region surrounding the highest hit within the cases. **e**. Figure representing the same block presented in (b) within the controls. **f**. Controls haplotype frequency, the red rectangle indicates the frequency across the controls of the most common haplotype identified within all the cases.
(TIF)

Figure S4 **Haplotype within the ∼2.5 Mb on chromosome E1.** ∼2.5 Mb are represented across all cases, controls and the two random bred cats excluded from the association study. The SNP in green across all samples represents the highest associated SNP with the disease. The orange bar represents the haplotype block shown in supplementary figure 2b and the relative position of the 2 candidate genes within the block is presented.
(PDF)

Table S1 **Evaluation of improved design to reduce genomic inflation (λ) in Burmese hypokalemia.**
(DOC)

Table S2 **Details of seven highest associated SNPs for Burmese hypokalemia GWAS.**
(DOC)

Table S3 **SNP analyses of *KCNH4* in cats with and without hypokalemia.**
(DOC)

Table S4 **Genotypes and frequencies of *WNK4* SNP in cats submitted for genetic testing.**
(DOC)

Table S5 ***KCNH4*** **and *WNK4* PCR primers for analysis of hypokalemia in cats.**
(DOC)
