# Supplementary figures and images for: First WNK4-Hypokalemia Animal Model Identified by Genome-Wide Association in Burmese Cats
Source: PLoS One. 2012 Dec 28;7(12):e53173. doi: 10.1371/journal.pone.0053173 (PMC3532348; doi:10.1371/journal.pone.0053173)

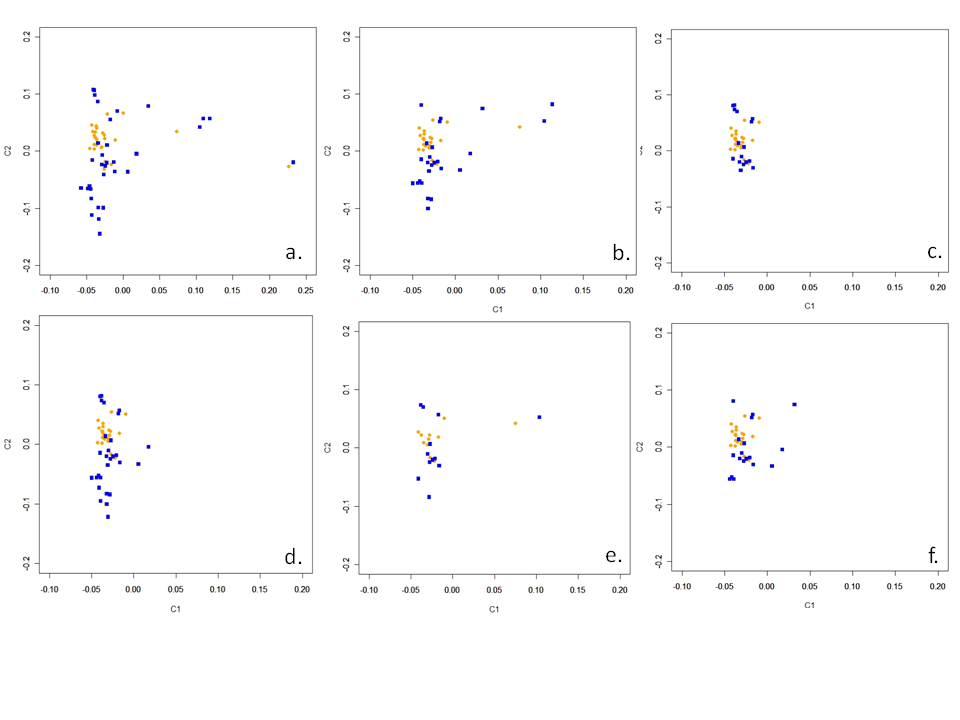

Supplement: Figure S1 — MDS plots illustrating the samples included in each case-control association analyses of cats with hypokalemia. MDS plots showing a. the distribution of all the genotyped samples, b. the samples included in the case-control after exclusion of the related Burmese cats (p_hat >0.3), c. the distribution of tightly clustered Burmese samples, d. allowing more diversity than the cluster illustrated in (c), e. the closest control for each case, and f. the samples remaining after exclusion of related Burmese and outliers. (TIF) [file pone.0053173.s001.tif]

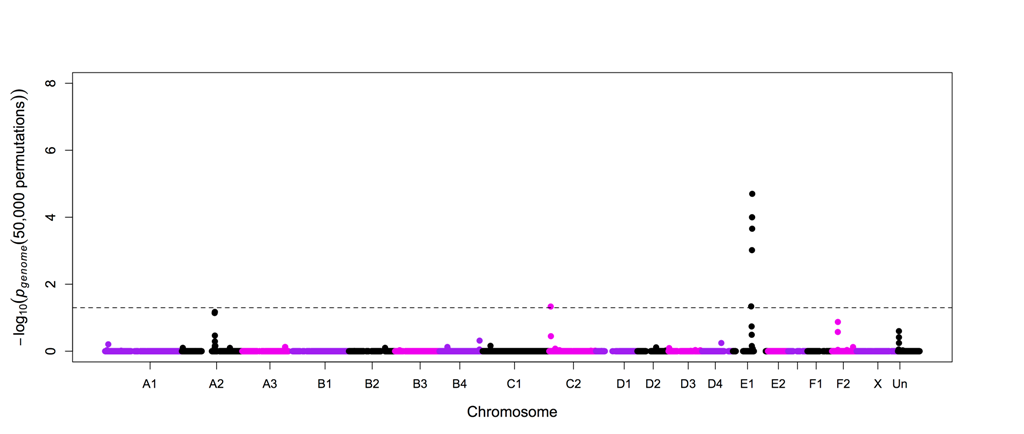

Supplement: Figure S2 — Manhattan plot of Burmese GWAS for hypokalemia. After correcting for multiple testing (50,000 permutations), five SNPs (four on chromosome E1 and one on chromosome C2) retained significant association (p<0.05; strongest SNP association, cat chromosome E1 position 73,054,644). The dashed line indicates genome wide significance. (TIF) [file pone.0053173.s002.tif]

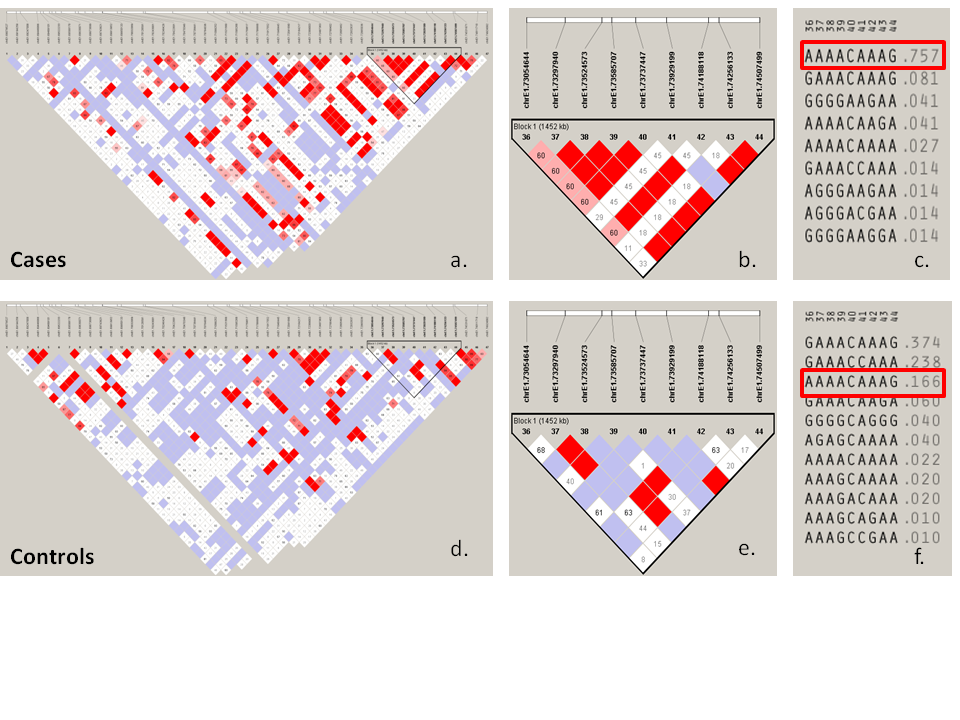

Supplement: Figure S3 — Haplotype analyses of cats in hypokalemia GWAS. Presented is the area within ∼2.5 Mb of the highest associated SNP on chromosome E2 in the Burmese cases and controls. a. Cases haplotype block of the region surrounding the highest hit in the Burmese. b. From the highest hit, a block extending for ∼1.45 Mb (9 SNPs) was identified c. Cases haplotype frequencies, the red rectangle indicates the haplotype most frequently found within the affected cats. d. Controls haplotype block of the same region surrounding the highest hit within the cases. e. Figure representing the same block presented in (b) within the controls. f. Controls haplotype frequency, the red rectangle indicates the frequency across the controls of the most common haplotype identified within all the cases. (TIF) [file pone.0053173.s003.tif]

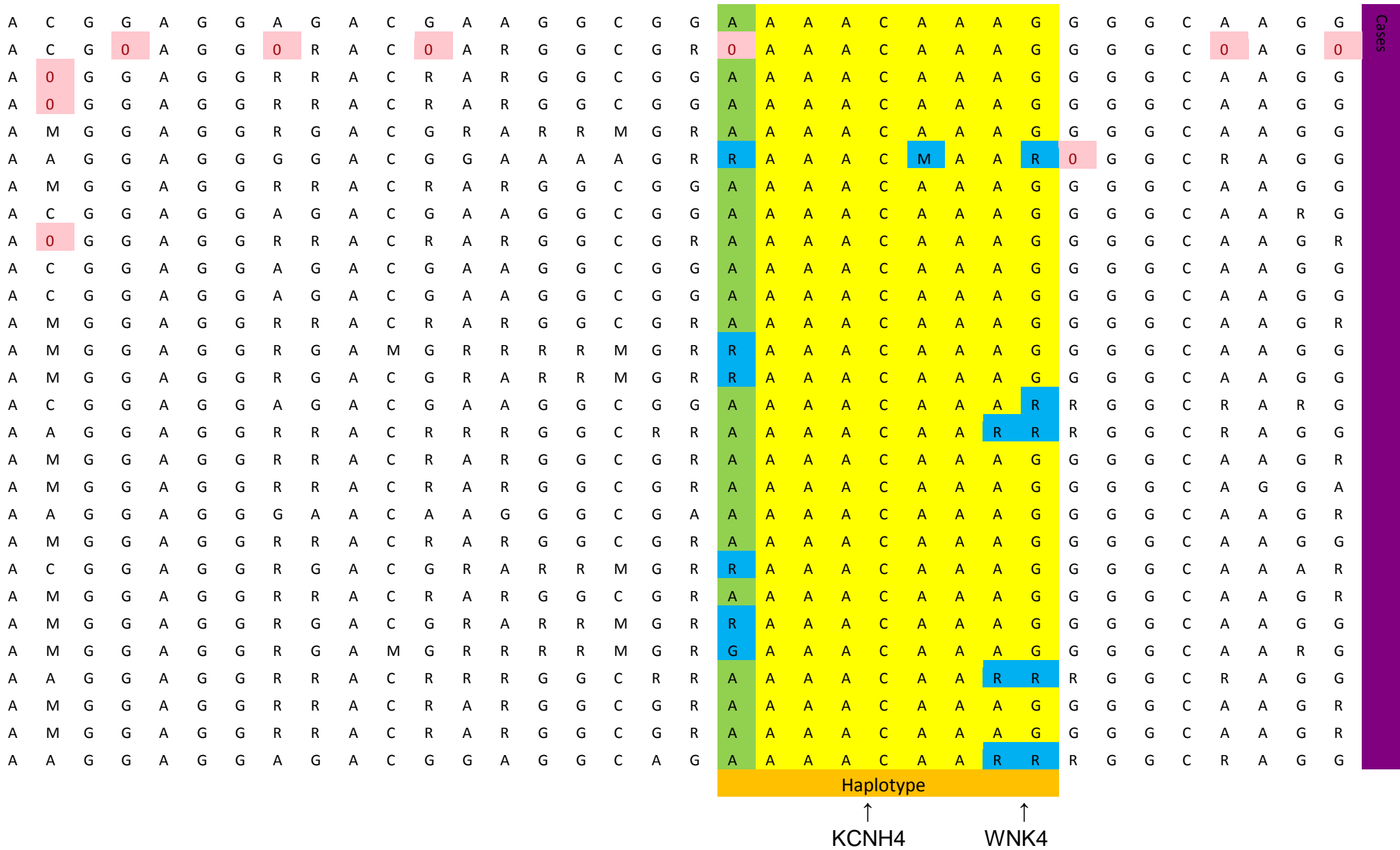

Supplement: Figure S4 — Haplotype within the ∼2.5 Mb on chromosome E1. ∼2.5 Mb are represented across all cases, controls and the two random bred cats excluded from the association study. The SNP in green across all samples represents the highest associated SNP with the disease. The orange bar represents the haplotype block shown in supplementary figure 2b and the relative position of the 2 candidate genes within the block is presented. (PDF) [file pone.0053173.s004.pdf]
