# Supplementary material for: First WNK4-Hypokalemia Animal Model Identified by Genome-Wide Association in Burmese Cats
Source: PLoS One. 2012 Dec 28;7(12):e53173. doi: 10.1371/journal.pone.0053173 (PMC3532348; doi:10.1371/journal.pone.0053173)
Supplement: Table S1 — Evaluation of improved design to reduce genomic inflation (λ) in Burmese hypokalemia. (DOC) [file pone.0053173.s005.doc]

**Table S1.** Evaluation of improved design to reduce genomic inflation (λ) in Burmese hypokalemia.

| **Method** | **Cases** | **Controls** | **Total** | **Genomic inflation** | **p-value** |
| --- | --- | --- | --- | --- | --- |
| P | 24 | 24 | 48 | 1.47 | 1.74x10^-8^ |
| Cluster 1 | 17 | 23 | 40 | 1.53 | 9.71x10^-7^ |
| Cluster 2 | 27 | 23 | 50 | 1.79 | 1.66x10^-9^ |
| Paired samples | 13 | 13 | 26 | 1.09 | 7.22x10^-5^***** |
| P+ Cluster 2 | 18 | 23 | 41 | 1.35 | 1.88x10^-6^ |

*The most significant SNP in the analysis is on chromosome E1 position 4497906 (6.59x10^-5^)
