# Supplementary material for: First WNK4-Hypokalemia Animal Model Identified by Genome-Wide Association in Burmese Cats
Source: PLoS One. 2012 Dec 28;7(12):e53173. doi: 10.1371/journal.pone.0053173 (PMC3532348; doi:10.1371/journal.pone.0053173)
Supplement: Table S2 — Details of seven highest associated SNPs for Burmese hypokalemia GWAS. (DOC) [file pone.0053173.s006.doc]

**Table S2.** Details of seven highest associated SNPs for Burmese hypokalemia GWAS.

| **SNP name** | **Chromosome** | **Old assembly position** | **Freq cases** | **Freq controls** | **p-value** |
| --- | --- | --- | --- | --- | --- |
| E1.73054644 | E1 | 69818460 | 0.882 | 0.291 | 9.27x10^-12^ |
| E1.72487363 | E1 | 69254825 | 0.886 | 0.3 | 1.29x10^-10^ |
| E1.72389003 | E1 | 69166616 | 0.871 | 0.26 | 1.31x10^-10^ |
| E1.72766402 | E1 | 69557010 | 0.871 | 0.3 | 5.13x10^-10^ |
| E1.72041690 | E1 | No blast | 0.814 | 0.26 | 3.65x10^-9^ |
| E1.69079527 | E1 | 66201518 | 0.929 | 0.54 | 6.99x10^-7^ |
| C2.5583090 | C2 | 5699657 | 0.797 | 0.333 | 7.35X10^-7^ |
