# Supplementary material for: First WNK4-Hypokalemia Animal Model Identified by Genome-Wide Association in Burmese Cats
Source: PLoS One. 2012 Dec 28;7(12):e53173. doi: 10.1371/journal.pone.0053173 (PMC3532348; doi:10.1371/journal.pone.0053173)
Supplement: Table S3 — SNP analyses of KCNH4 in cats with and without hypokalemia. (DOC) [file pone.0053173.s007.doc]

**Table S3**. SNPs analyses of *KCNH4* in cats with and without hypokalemia.

| **Breed** | **Phenotype** | **c.T2563G** | **c.T2663G** | **c.T2724C** | **c.G2759A** | **-26E2 A>G** | **+38E2 A>G** | **+15E9 CC>-** | **-34E12 C>T** |
| --- | --- | --- | --- | --- | --- | --- | --- | --- | --- |
| Burmese | Case | T | G | T | G | A | A | CC | C |
| Burmese | Case | T | G | T | G | A | A | CC | C |
| Burmese | Control | T | G | T | G | A | A | CC/- | C |
| Burmese | Control | T | G | T | G | A | A | CC | C |
| Random Bred | Control | T | K | Y | G | R | R | CC/- | Y |
| Trace | - | G | G | C | A | - | A | CC | C |
| AA | change | V ->G | - | S -> P | - |  |  |  |  |
