# Supplementary material for: First WNK4-Hypokalemia Animal Model Identified by Genome-Wide Association in Burmese Cats
Source: PLoS One. 2012 Dec 28;7(12):e53173. doi: 10.1371/journal.pone.0053173 (PMC3532348; doi:10.1371/journal.pone.0053173)
Supplement: Table S4 — Genotypes and frequencies of WNK4 SNP in cats submitted for genetic testing. (DOC) [file pone.0053173.s008.doc]

**Table S4.** Genotypes and frequencies of *WNK4* SNP in cats submitted for genetic testing*.

| Breed | No. |  | Genotype |  | T allele |
| --- | --- | --- | --- | --- | --- |
|  |  | C/C | C/T | T/T | Frequency (%) |
| Asian | 9 | 6 | 3 | 0 | 16.6 |
| Australian Mist | 2 | 2 | 0 | 0 | 0 |
| Burmese | 11 | 0 | 0 | 11 | 15.5 |
|  | 234 | 180 | 54 | 0 |  |
| Burmilla | 3 | 3 | 0 | 0 | 0 |
| Tiffanie | 7 | 6 | 1 | 0 | 7.1 |
| Unknown | 2 | 2 | 0 | 0 | 0 |
| Total | 268 | 199 | 58 | 11 | 14.9 |

*Testing performed at Langford Veterinary Services
