# Supplementary material for: First WNK4-Hypokalemia Animal Model Identified by Genome-Wide Association in Burmese Cats
Source: PLoS One. 2012 Dec 28;7(12):e53173. doi: 10.1371/journal.pone.0053173 (PMC3532348; doi:10.1371/journal.pone.0053173)
Supplement: Table S5 — KCNH4 and WNK4 PCR primers for analysis of hypokalemia in cats. (DOC) [file pone.0053173.s009.doc]

**Table S5**. *KCNH4* and *WNK4* PCR primers for analysis of hypokalemia in cats.

|  |  | | |  | | ***KCNH4* Genomic Primers** | | |  | | |
| --- | --- | --- | --- | --- | --- | --- | --- | --- | --- | --- | --- |
| Exon | Product Size (bp) | | | Mg^2+^/⁰C | | Forward Primer 5’-3’ | | | Reverse Primer 5’-3’ | | |
|  |  | | |  | |  | | |  | | |
| 2 | 561 | | | 1.5/65 | | GGACCCAGGAATCAAATGAAGCC | | | CTGACTCCTTCTCTTCCAGATGGG | | |
| 3-4 | 671 | | | 1.5/65 | | CCCATCTGGAAGAGAAGGAGTCAG | | | ACGGGAGGGAGATCAGATAGGCA | | |
| 5 | 521 | | | 1.75/63 | | GCACCTTCTATCTCCTGTCGTGGT | | | GGGCACATGGACCATTAGGACTTAA | | |
| 6 | 662 | | | 1.75/63 | | TTAAGTCCTAATGGTCCATGTGCCC | | | CCGCCTTGGTAGCCCTAGTTAGC | | |
| 7 | 490 | | | 1.75/63 | | GTCACTTTCTCAGGACAGCAGGGA | | | TCTTCACTTCAGTACCCCCGTGC | | |
| 8 | 390 | | | 1.5/67 | | CAGGGAAGGCCAGCTCTCCACT | | | AACGGCTATGAATGAATGCACGA | | |
| 9 | 524 | | | 1.5/67 | | GAGAGGAACATCAGGTCTGGCTATTC | | | CAAGGAAAAGGGATGTGGATGGG | | |
| 10 | 457 | | | 1.75/63 | | GTCAAACACAGGTAACTTCAAAGCAA | | | GGTATCCCAGGCCCTAGATTCC | | |
| 11 | 527 | | | 1.75/63 | | CCAACTCCATTGATTATTTCCACCA | | | GCCGGGGATTGAGAAATGATGTTCT | | |
| 12 | 285 | | | 1.5/65 | | AGACTTACTTTGCCACTCAGACATCTC | | | AAGGTGCCTCCAGCTCCCAGAC | | |
| 14 | 481 | | | 1.75/63 | | CCCTTCCTGATCCACCACACTTG | | | GTTCTGGAATCGTTTATCTGAGGACAC | | |
| 15 | 404 | | | 1.75/63 | | AGGAGAGAAGAGCATGTTGTGTTCAG | | | GATGAGGAAATTGAAGTTTGGGGTG | | |
| 16 | 824 | | | 1.5/65 | | CGGTGATTGTTGTGGCATCTGG | | | TCTTGCCTCAGTCTTCGTGGGCT | | |
|  | |  |  | | ***WNK4* Genomic Primers** | | |  | | | |
|  | |  |  | |  | | |  | | | |
| 1 | | 756 | 1.5/65 | | TGTCAGACCGCCTCCTCTCCAG | | | GCTGCTGTCCCCTAGGTTTCTCAT | | | |
| 2 | | 307 | 1.5/65 | | CCACAGACTCGGAAACTCTCTCG | | | GGGTGAGAGCCTGATTTTGGAC | | | |
| 3 | | ~ 1000* | 1.5/65 | | TTCCCGCAGTCTGGTCAGCCTTTCTCC | | | GATGCCATAAGTCTCCCCACTTTCC | | | |
| 4 | | 787 | 1.5/65 | | CCGAGAGGAAAGAGGAAGCCAGA | | | CACCTCTCGTTCTTATCTGTGCGGAT | | | |
| 5-6 | | 569 | 1.5/65 | | CTAGGGCACAAAGCCGAACAGC | | | TTGGGGCGTGCGTGTGTTTAG | | | |
| 7 | | 433 | 1.5/65 | | TCACCTCAAACCGTGCTCCTCG | | | TCGTGGCTAGTTGTGGAAGGCAG | | | |
| 8-9-10 | | 756 | 1.5/65 | | TGTCACTTACCCTGCCTTCCACAAC | | | CCTGACAGTTCCCTCCTGGTCCC | | | |
| 11 | | 397 | 1.5/65 | | TTTCTAATATCAGACTTTGGGGGCA | | | ATGTCTTCAGTGTCATCATGCTTTAAA | | | |
| 12 | | 380 | 1.5/65 | | TTTAAAGATCAGTGCAGGGGAGAGA | | | TGGCTTTCATCAGATACTCAAATGG | | | |
| 13 | | ~ 310* | 1.5/65 | | CCATTTGAGTATCTGATGAAAGCCA | | | ATCCCTAAGCCCTTCCCAGGAG | | | |
| 14-15 | | 855 | 1.5/65 | | CTCCTGGGAAGGGCTTAGGGAT | | | AGAAATTGGCAGGGTATTAAGTGGAAG | | | |
| 16 | | 515 | 1.5/65 | | TGCCACCTTCCACTTAATACCCTG | | | CAAGTCCACCTCTTATCCTTCCTCC | | | |
| 17-18 | | 1115 | 1.5/65 | | GGAGGAAGGATAAGAGGTGGACTTG | | | AGCACTGACGAAACCTTCAGGATG | | | |
| 19 | | 420 | 1.5/65 | | CATCCTGAAGGTTTCGTCAGTGCT | | | TCACAGATTCCTAGTTTTCGGCATTT | | | |
|  | |  |  | |  | | |  | | | |
|  | | |  | | | | **cDNA Primers** | | |  |  |
| Primer name | | | Mg/T | | | | Forward Primer 5’-3’ | | |  |  |
|  | | |  | | | |  | | |  |  |
| WNK4-5utr-F | | | 1.5/65 | | | | GCCTGTCAGACCGCCTCCTC | | |  |  |
| WNK4-5utr-R | | |  |  |  |  | GCCGCTCCGTTCGAGAGAGTTT | | |  |  |
| WNK4- 1F | | | 1.5/65 | | | | AAACTCTCTCGAACGGAGCGGC | | |  |  |
| WNK4- 1R | | |  |  |  |  | GTACTCTGAGGTAGCCATCTCCAGCA | | |  |  |
| WNK4- 2F | | | 1.5/65 | | | | TGTACGAGGAAAAGTACGATGAGGC | | |  |  |
| WNK4- 2R | | |  |  |  |  | ATGGCAGCAACTCGTTCACGTACT | | |  |  |
| WNK4- 3F | | | 1.5/65 | | | | CAGATAAGAACGAGAGGTTCACCATCC | | |  |  |
| WNK4- 3R | | |  |  |  |  | GCATCTGAGGCATAGCTGTCTCCC | | |  |  |
| WNK4- 4F | | | 1.5/65 | | | | GATTGCGAGACTGATGGCTACCTC | | |  |  |
| WNK4- 4R | | |  |  |  |  | GAAAACAGGCTTCCAGGAGACAAG | | |  |  |
| WNK4- 5F | | | 1.5/65 | | | | GAGACCCTGTTGAAGAGAGATACTGG | | |  |  |
| WNK4- 5R | | |  |  |  |  | TGTGGCTTTCCCTCTTCAGAGATG | | |  |  |
| WNK4- 6F | | | 1.5/65 | | | | CCTTCTCACTGGCTGTGATGACTGT | | |  |  |
| WNK4- 6R | | |  |  |  |  | CTCCGACAAGTGCTTCTGCCGAA | | |  |  |
| WNK4- 7F | | | 1.5/65 | | | | AGTGTGGATGAACTACTCATACAGCAGC | | |  |  |
| WNK4- 7R | | |  |  |  |  | ATTCACATCCTGCCAACATCCCC | | |  |  |
| WNK4- 8F | | | 1.5/65 | | | | GGCATCATGCGAAGGAACTCC | | |  |  |
| Poly(T) | | |  |  |  |  | Invitrogen | | |  |  |
| Hypo For  Hypo Rev  Hypo Seq | | |  | | | | CTGCTCCCCTCCCTAGCTT  Biotin-CTCTCGGTCTCAGCTGTTGG  CTCCCCCTGCTCCTTGTGGC | | |  |  |

* The length of the PCR product could not be determined exactly, due to incomplete overlap of forward and reverse sequence.
